# Sacral Dural Tears as a Cause of Spontaneous Intracranial Hypotension

**DOI:** 10.1007/s00062-023-01292-0

**Published:** 2023-06-01

**Authors:** Niklas Lützen, Enrique Barvulsky Aleman, Amir El Rahal, Florian Volz, Christian Fung, Jürgen Beck, Horst Urbach

**Affiliations:** 1 Dept. of Neuroradiology, Medical Center—University of Freiburg, Faculty of Medicine, University of Freiburg, Breisacher Str. 64, 79106 Freiburg, Germany; 2 Dept. of Neurosurgery, Medical Center—University of Freiburg, Faculty of Medicine, University of Freiburg, Breisacher Str. 64, 79106 Freiburg, Germany

**Keywords:** Cerebrospinal fluid leak, Epidural blood patch, Orthostatic headache, Computed tomography myelography, Magnetic resonance imaging

## Abstract

**Purpose:**

Dural tears at the level of the cervical, thoracic, and lumbar spine cause spontaneous intracranial hypotension (SIH) in patients with a spinal longitudinal extradural CSF collection (SLEC); however, sacral dural tears have rarely been reported so far. This study focuses on sacral dural tears as a cause of SIH.

**Methods:**

Retrospective data from SIH patients with confirmed sacral dural tears studied between October 2020 and November 2022 were analyzed with respect to demographic, clinical and imaging features. Digital subtraction myelography (DSM) and lumbar epidural blood patch (EBP) were modified by placing the patient in reversed Trendelenburg position.

**Results:**

Of the SIH patients, 9 (all women; mean age, 38.5 years; mean body mass index, BMI, 22.9) out of 149 had a sacral dural leak (6%) that occurred spontaneously in 7/9, while 2/9 were likely associated with minor trauma. None had a sacral fracture. The mean SIH score was 6.8. All patients showed SLEC on heavily T2-weighted MR myelography (T2-MRM), 4/9 exclusively sacral and 5/9 with partial or complete involvement of the remaining spine. 4/9 had none, but 5/9 had meningeal sacral cysts, 2/5 had large cysts/ectasia. Confirmation of the sacral origin of the leak was provided in 4/9 by T2-MRM, in 2/9 by DSM and 3/9 by CT myelography (CTM) whereas 0/9 revealed the exact site of leak within the sacrum.

**Conclusion:**

Sacral dural tears should be considered as a possible cause for SIH. It is concluded to implement T2-MRM covering the entire sacrum in the standard MRI protocol and propose EBP in the reverse Trendelenburg position as a therapeutic approach.

## Introduction

Spontaneous intracranial hypotension (SIH) is an acquired disorder usually caused by spinal CSF leaks. The key symptom is orthostatic headache, which may be accompanied by various symptoms with coma as the most serious presentation [1, 2]. Patients who show a spinal longitudinal extradural CSF collection (SLEC) on imaging have an underlying dural tear at the ventral, lateral [3] or dorsolateral [4] aspect of the spinal thecal sac. Patients that are SLEC-negative often exhibit a CSF venous fistula. The exact site of leakage in SLEC-positive patients ranges from the lower cervical to the lumbar spine, with predominant thoracic localization [3, 5].

Schievink et al. [4] also considered complex cysts/dural ectasia as a cause of SIH in their classification system for spinal CSF leaks. While these cysts often occur at the sacral level [4, 6, 7] it is not further specified whether they are associated with an SLEC. There are single case reports in the literature in which a tear of a sacral meningeal cyst was considered the cause of a CSF leak, most of them with a history of a trauma [8–12]. Some others reported on patients with a CSF leak in the presence of an underlying sacral dural ectasia or anterior sacral meningocele, [13, 14] that can occur, e.g., in Marfan’s syndrome; however, these reports usually did not demonstrate the sacral origin of the CSF leak.

While most dural leaks in SLEC-positive SIH patients can be accurately identified, 10% or more cases remain undetected [3, 4]. Sacral dural tears as a cause of spontaneous intracranial hypotension in SLEC-positive patients may close this diagnostic gap.

The purpose of this study was to evaluate sacral dural tears in SLEC-positive SIH patients and to highlight specific imaging and demographic findings. We also present approaches for the diagnostic work-up and treatment of patients with sacral leaks.

## Material and Methods

### Data Selection and Review

The local ethics committee approved the trial (vote number: 22-1249-S1-retro). Written informed consent was obtained from each patient.

The study population consisted of 149 patients with the diagnosis of SIH, according to the International Classification of Headache Disorders, 3rd edition [15] investigated between October 2020 and November 2022. Inclusion criteria for this retrospective study were the following: 1) presence of a SLEC on spinal MR imaging, 2) confirmation of the sacral origin of the leakage by T2-MRM, DSM or CTM.

Demographic data of included patients were extracted from the patient’s charts and included: sex, age, body mass index (BMI) and a trauma history as a possible trigger of SIH. Clinical data such as initial symptoms and changes in symptoms at follow-up were recorded. Therapeutic approaches performed at our institution for patients with a sacral leak were analyzed.

**Fig. 1:**
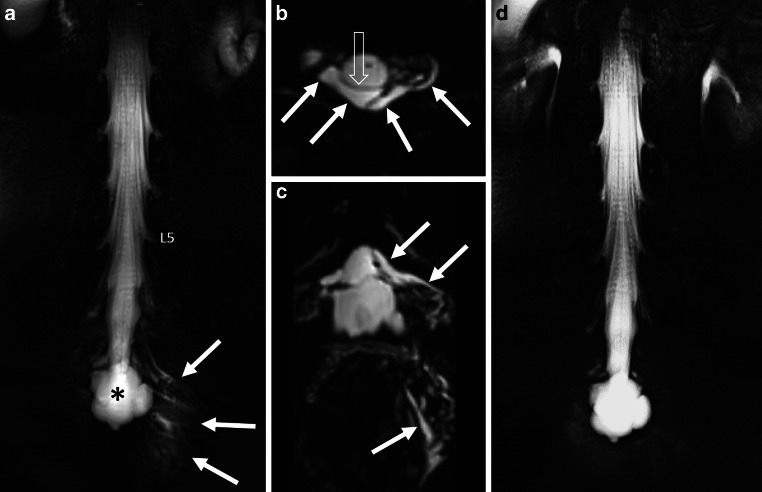
34-year-old woman with a sacral dural leak and SIH score of 7. Spinal longitudinal extradural CSF collection (SLEC) is exclusively visible at the sacral level on coronal 2D heavily T2-weighted MRI (T2-MRM) (*arrows* in **a**) and on axial and coronal 3D T2-MRM (*arrows* in **b**, **c**), representing the sacral origin of leak. The SLEC is well distinguishable from the dura (*open arrow* in **b**). A large meningeal cyst/dural ectasia is visible on coronal 2D T2-MRM (*asterisk* in **a**). After lumbar epidural blood patch (EBP) in reversed Trendelenburg position, SLEC completely resolved (**d**)

Images were reviewed by two radiologists with 8 (CAQ-certified in neuroradiology) and 5 years of experience in neuroradiology based on consensus:

- Calculation of the established Bern SIH score [16] on head MRI (a 9-point Likert-type scale indicating the probability for the presence of SIH as 0–2 points to be low, 3–4 points to be intermediate and ≥ 5 points to be high).
- Presence of a SLEC on heavily T2-weighted MR images (2D T2-weighted sequences (Fig. 1a) and 3D isotropic T2-weighted fat-saturated sequences (Fig. 1b,c)).
- Presence of sacral meningeal cysts (< 3 cm in diameter) or complex sacral cysts/ectasia (defined to be larger than 3 cm).
- Indications of a sacral fracture on MRI or CT.
- Confirmation of the sacral origin of the dural tear on MRI (defined as the presence of a SLEC occurring exclusively at the sacral level), DSM or CTM (visible sacral extradural contrast outflow).
- Exact localization of sacral dural tear.
- Resolution of the SLEC on MRI, DSM or CTM after treatment, if available.

Demographic, clinical and imaging findings are listed in Table 1. The MRI parameters for heavily T2-weighted MR-myelography images are listed in Table 2.

**Table 1:** Demographic characteristics and MR findings of included patients

Nr	Age (years)/ gender	BMI	Initial symptoms	History of trauma	SIH Score	SLEC on 3D T2-MRM	SLEC on 2D T2-MRM	Sacral cysts	Exact site of leakage	Treatment	SLEC after treatment	Symptoms after treatment (time to clinical follow-up)
1	32 F	31.2	Orth. headache, nausea	No	9	Yes (S, C, T, L)	No	< 3 cm	S2 or S3 left	2 × fibrin	RV	1 (< 3 months)
2	25 F	22.6	Orth. headache, nausea, vomiting	No	6	Yes (S)	Yes	No	N/A	1 × EBP	N/A	3 (> 9 months)
3	56 F	19.6	Orth. headache, neck pain	Yes (yoga)	7	Yes (S, C, T, L)	N/A	< 3 cm	No	4 × EBP, 1xfibrin	No	3 (> 18 months)
4	34 F	19.6	Orth. headache	No	7	Yes (S)	Yes	> 3 cm	N/A	3 × EBP	No	2 (> 6 months)
5	26 F	24.0	Orth. headache, neck pain, dizziness	No	4	Yes (S)	Yes	No	N/A	1 × EBP	N/A	1 (> 3 months)
6	40 F	19.7	Orth. headache	No	6	Yes (S)	Yes	No	No	2 × EBP	No	3 (< 3 months)
7	36 F	21.8	Orth. headache	No	7	Yes (S, C, T, L)	Yes	No	S2 or S3 right	1 × EBP	Yes	2 (> 3 months)
8	40 F	21.3	Orth. headache	No	7	Yes (S, L)	Yes	> 3 cm	No	1 × EBP	No	3 (> 12 months)
9	59 F	26.3	Headache, visual impairment, tinnitus	Yes (fall on back)	8	Yes (S, L)	No	< 3 cm	S2 or S3 left	2 × EBP	Yes	0 (< 3 months)

*Symptoms after treatment: 0 (unchanged), 1 (improved), 2 (predominantly improved), 3 (completely resolved), 4 (worsened)*

*Orth. headache* orthostatic headache,* C* cervical,* T* thoracic,* L* lumbar,* S* sacral,* RV* residual volume,* N/A* not available

**Table 2:** 1.5 T MRI parameters for heavily T2-weighted MR images (2D and isotropic 3D T2-weighted sequences)

Sequences	2D MR-myelography (T2 HASTE)	3D MR-myelography (T2 SPACE fs)
Orientation	Sagittal + coronal	Sagittal
TR (ms)	1100	1500
TE (ms)	10,000	226
FA (°)	180	130
FOV (mm)	100	100
Matrix	448 × 448	320 × 320
Voxel size (mm)	60	0.74
Sections	1	60
TA (min:s)	0:20	6:10

*HASTE* half fourier-acquired single shot turbo spin,* SPACE* sampling perfection with application-optimized contrasts by using different flip angle evolutions,* TR* repetition time,* TE* echo time,* FA* flip angle,* FOV* field of view,* TA* acquisition time

### Imaging Techniques

**Fig. 2:**
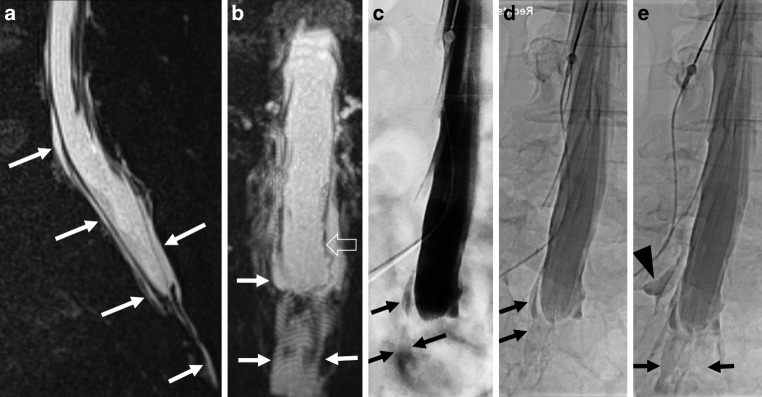
36-year-old woman with a sacral leak and SLEC extending from sacral to cervical level (not shown) with an SIH score of 6. 3D T2-MRM shows SLEC in sagittal (**a**) and coronal (**b**) orientation without evidence for an underlying sacral meningeal cyst. Dura is well distinguishable from SLEC (*open arrow* in **b**). Digital subtraction myelography (DSM) in right lateral decubitus position in anterior-posterior projection shows extradural contrast outflow (*arrows* in **c**), that increases from level S2 right side to caudal over time (*arrows* in **d**, **e**) with additional contrast outflow at level S1 right (*arrowhead* in **e**), representing sacral origin of leak

The DSM was performed on a flat detector unit (Siemens icono biplane, Siemens Healthineers, Erlangen, Germany) with the patient in the lateral decubitus position (on the side where the leak most likely occurred on spinal MRI) in monoplane or biplane acquisition. After lumbar puncture with a 20-ga spinal needle, the table was tilted with the upper body of patient elevated 6–7° (reversed Trendelenburg position) (Fig. 2c–e, 3c,d), a modified technique that has recently also been described for the detection of sacral CSF-venous fistulas [17]. Subsequently, 10–15 ml of contrast agent (300 mg iodine/ml; Iomeprol 300M, Bracco, Switzerland) was injected intrathecally. Still placed in the lateral decubitus position, patients were transferred to the CT scanner.

**Fig. 3:**
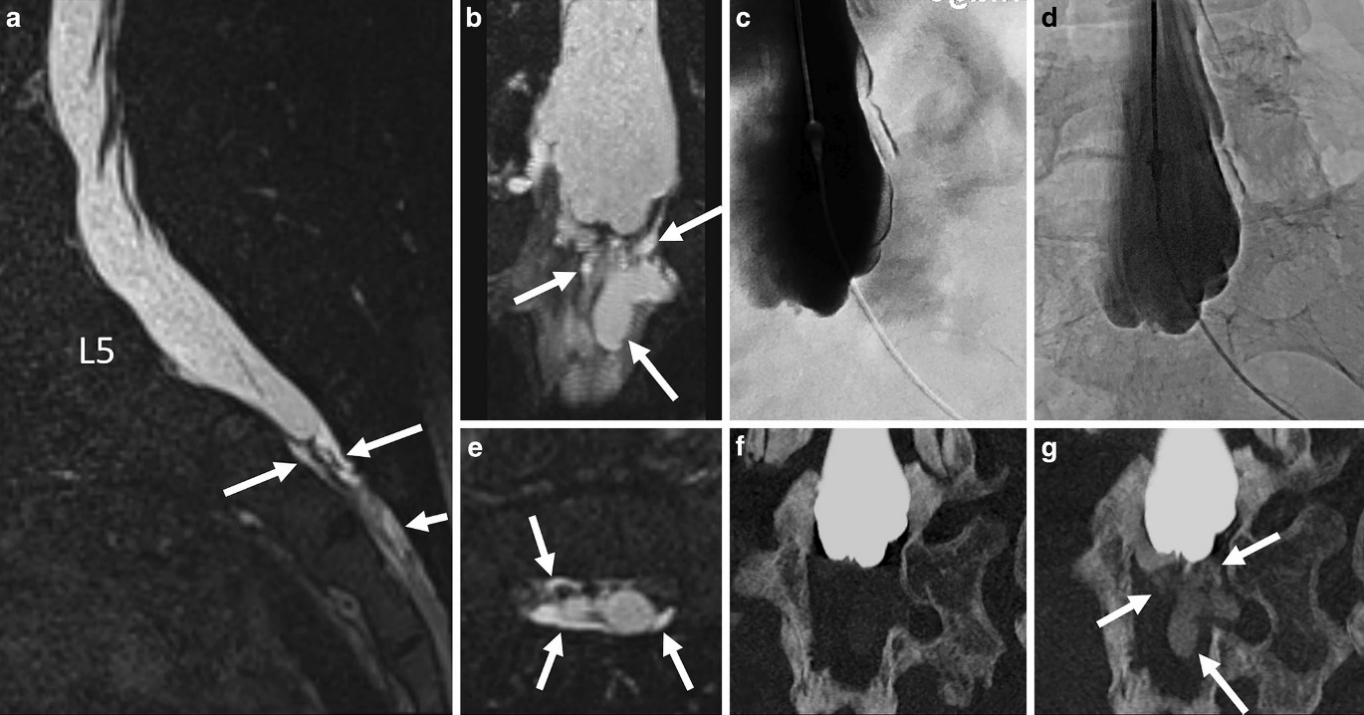
40-year-old woman with a subtle sacral leak and SIH-score of 6. Sagittal, coronal and axial 3D T2-MRM show a small SLEC at the sacral level (*arrows* in **a**, **b**, **e**). Linear fluid indicates the extradural component of SLEC (*arrows* in **e**). Neither DSM in left lateral decubitus and reversed Trendelenburg positions(**c**) nor delayed single X‑ray in anterior-posterior projection reveal contrast outflow (**d**). Coronal CT myelography in supine position does not show epidural contrast outflow with certainty (**f**) but delayed CT scan 30 min after walking around does (*arrows* in **g**), representing the sacral origin of leak

All CTs were performed on a 64-row multidetector CT scanner (Somatom Definition 64 AS; Siemens Healthineers). Following DSM, the CT scan in the lateral decubitus or supine position ranged from lumbar puncture to the entire sacrum. The CT scan was planned with caution regarding the female reproductive organs, which were excluded from the field of examination whenever possible, because of the average young patient age. If epidural sacral contrast leakage could not be demonstrated with certainty, we performed a delayed supine CT scan in a few cases after the patient walked around for 20–30 min to provoke extradural contrast outflow (Fig. 3f,g).

### Treatment Techniques

All lumbar epidural blood patches (EBP) were performed on a flat detector unit (Siemens icono biplane, Siemens Healthineers) in the lateral decubitus position (on the side where the leak most likely occurred). A 20-ga spinal needle was placed into the epidural space, preferably performed at the lower lumbar segments. Correct epidural position was confirmed by a minimal amount of iodine contrast (about 1–2 ml). Thereafter, the table was tilted 6–7° so that the patient was placed in a reversed Trendelenburg position to support the epidural blood flow towards the sacrum. To the best of our knowledge, this modified technique has not been described previously in context with an EBP. A minimum of 20 ml and a maximum of 50 ml of the patient’s own blood was administered. This technique was primarily used when the location of the leak within the sacrum could not be more accurately visualized; however, it can also be used as a first therapeutic measure, as it is easy and straightforward to apply.

The CT-guided fibrin patches were performed with the patient in a prone position on a 16-row multidetector CT scanner (Somatom Scope; Siemens Healthineers). This technique may be applied if it is possible to narrow down the site of leak to a certain segment. A 20-ga spinal needle was placed into a sacral cyst or epidurally next to a nerve root sleeve suspicious for the sacral leak. A minimal amount of contrast medium was injected to confirm the correct position and 2 or 4 ml of fibrin was introduced (Tisseel 2ML, Baxter Deutschland GmbH, Germany).

Therapeutic success was defined radiologically and clinically. On MRI follow-up, resolution of the SLEC was considered successful treatment; predominantly decreased SLEC was considered as therapeutic response requiring more EBP and constant SLEC was considered unsuccessful treatment (Table 1). The clinical assessment was divided by changes of symptoms: unchanged, improved, predominantly improved, completely resolved, worsened (Table 1).

## Results

### Patient Characteristics

**Fig. 4:**
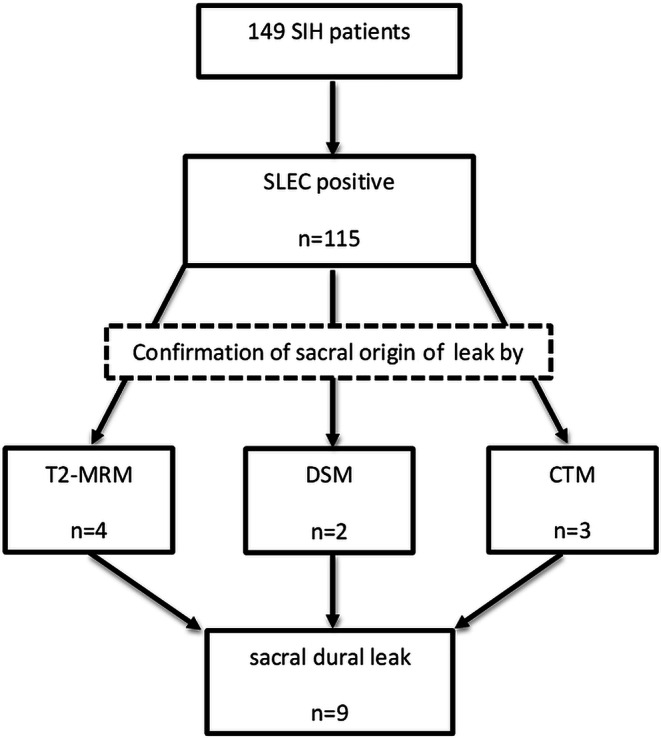
Flowchart of patient selection

Out of 149 SIH patients 9 (6%, all women) had a confirmed sacral dural tear and were enrolled into the final analysis (Fig. 4). The mean age was 38.5 years (range 25–59 years) and mean BMI was 22.9 (range 19.6–32.1). One patient (# 9) stated that she had fallen on her back before the onset of symptoms. Another patient (# 3) stated that she practiced yoga, which was temporally related to her symptoms.

### Imaging Findings

The initial mean Bern SIH score was 6.8 (range 4–9), 3/9 had hygromas and 1/9 presented with tonsil herniation. In 9/9 patients SLEC was visible on 3D isotropic heavily T2-weighted MR images with fat saturation (e.g., Fig. 1b,c). In 4/9 patients SLEC was exclusively seen in the sacral region on MRI (Fig. 1a–c), in 2/9 patients SLEC was seen in the sacral region with involvement of the lumbar region (Fig. 2a) and in 3/9 patients SLEC was visible from sacrum to cervical spine on 3D T2-MRM. 8/9 patients received coronal and sagittal 2D heavily T2-weighted MR images that has been implemented into our standard MRI protocol since September 2021. On these 2D T2-MRM, evidence of sacral SLEC was visible in 6/8 patients (Fig. 1a).

Small meningeal sacral cysts were delineable in 3/9 patients (< 3 cm in diameter), whereas large meningeal cysts (> 3 cm in diameter) occurred in 2/9 having the appearance of a dural ectasia (Fig. 1). In 4/9 patients, no meningeal cysts were visible at sacral level on MRI, DSM or CTM, if available (Fig. 2).

In 4/9 patients, the diagnosis of a sacral origin of the leak could be made by 3D T2-MRM (due to an exclusive manifestation of a SLEC at the level of the sacrum). In 2/9 patients the diagnosis of sacral origin was made by DSM (Fig. 2c–e) and in 3/9 patients by CTM (Fig. 3f,g) (1 patient demonstrated SLEC exclusively at the sacral level on CTM, 2 patients revealed an increasing contrast filling of the extradural sacral compartment in an additional delayed CT scan). In 3/9 patients the dural tear could be narrowed down to the level of S2 or S3 but the exact site of leak within the sacrum could not be shown with certainty in any case.

None of the patients showed evidence for a sacral fracture (in 6/9 patients a CT scan and in 3/9 MRI was available to search for a sacral fracture).

### Therapeutic Approaches and Preliminary Results

Out of 9 patients with a confirmed sacral leak, 8 received at least one epidural blood patch (EBP) at the lower sacrum with the patient in reversed Trendelenburg position. 4/9 had multiple EBPs (range 2–4) and 2/9 had a targeted CT-guided fibrin (range 1–2) patch where the leak most likely occurred.

So far, seven patients had follow-up MRI after treatment. In 4/7 the SLEC completely resolved after EBP (three patients had multiple, one patient had a single EBP) within reversed Trendelenburg position. In one patient two CT-guided fibrin patches showed therapeutic response with predominantly decrease of the SLEC (residual volume). Two patients did not respond to EBP (range 1–2) so far (constant SLEC).

All patients had clinical follow-up, 6/9 in a period >3 months after the last treatment (range > 3–> 18 months). 3/9 had clinical follow-up in a period <3 months, which is a limitation. A good clinical outcome could be noted in 6/9 patients (symptoms predominantly improved or completely resolved). Two patients had a mild to moderate response, one patient has not clinically improved to date.

## Discussion

In this study, we demonstrate an underlying sacral dural leak as a cause of SIH in 9 out of 149 patients (6%).

All patients with a sacral leak in this cohort were women, which is notable. In the literature, we found a single case report of a man in whom a rupture of a sacral Tarlov cyst was considered the cause of SIH [12]. Further studies have yet to show whether women with sacral leaks are more than twice as likely as men to be affected, as is usually the case with SIH [18]. The average age of patients in our cohort was 38.5 years and the average BMI was 22.9, which is slightly lower than in the common SIH cohort (38.5 vs. 45.7 years, 22.9 vs. 26) [4, 5].

All patients in our cohort had distinct signs of SIH on head MRI, assessed by the established Bern SIH score [16]. In 8/9 patients the initial score was ≥ 5 points (high probability for SIH) and 1/9 had an initial score of 4 (intermediate probability for SIH). In all patients SLEC was visible on 3D T2-MRM, which has already been shown to reliably demonstrate a SLEC in SIH patients as sensitive as gadolinium-enhanced MR myelography [19] or CT myelography [20]. Once the first patient with a sacral dural leak was identified at our institution, routine MRI examinations of SIH patients were performed with consistent coverage of the entire sacrum. In our experience, the sacrum is often only partially covered on spinal MRI, as it is generally not considered important. Moreover, 2D T2-MRM is also of value, in our study they could show sacral involvement of a SLEC at a glance in 6/8 cases (Fig. 1a).

In the literature, sacral dural tears are generally not assumed as a cause of SIH [21]. In their classification system, Farb et al. [3] did not report on sacral leaks whereas Schievink et al. [4] considered complex cysts/dural ectasia as a cause of SIH (in 4% of all SIH patients). They described them most often to occur at the sacral level, but did not specify whether these have a SLEC. There are a few cases in the literature reporting on intracranial hypotension most likely due to a sacral CSF leak. Most of them reported underlying trauma, except two cases [12, 22]; however, all cases were associated with sacral meningeal cysts, which are a potential expression of dura weakness: Tarlov cysts [8–11], dural ectasia or anterior sacral meningoceles [13, 14]. Tarlov cysts are CSF-filled sacs within the nerve root sleeves, that usually occur at the sacral level of the spine and only rarely appear with symptoms by compression of spinal nerve roots [23]. Ruptured sacral Tarlov cysts are most likely different from spinal lateral CSF leaks type 1b according to Schievink et al. [7], which is most likely a dural tear in the region of a nerve root sleeve with an arachnoid outpouching, appearing like a meningeal cyst [24]. Dural ectasia is a ballooning of the thecal sac, which most often occurs in the sacrum and can be present in several genetic disorders most commonly in Marfan’s syndrome [6]. Dural ectasia and the severe form of anterior sacral meningocele, which extends through the ventral cortex of the sacrum, may even cause SIH without a dural tear by pooling the CSF [6].

In our cohort, 4/9 patients did not show any sacral cysts, a condition previously not reported with sacral leaks. Consequently, a causative dural weakness in these patients is less likely. Moreover, 7/9 patients in our study had a sacral tear without history of trauma while 2/9 had only minor trauma or yoga lessons and there was no fracture. Therefore, we consider a traumatic origin of sacral leaks as unlikely.

Neither the exact location within the sacrum nor the precise anatomic nature of the leak could be demonstrated in each case in our study. This could be due to the complex anatomy of the sacrum and, as we observed, the often slowly filling meningeal cysts. Microsurgical exploration may help to identify the nature of the leak as well as histological analysis. It is reasonable, however, not to choose a surgical approach in the first line treatment if the site of the leak within the sacrum is not exactly identifiable. Moreover, as reported in surgery of symptomatic Tarlov cysts, complication rate may be high [25]. We decided to perform an EBP at the lower lumbar site with the patient in the reverse Trendelenburg position. On MRI follow-up, 5/9 patients showed response to EBP/CT-guided fibrin patch, whereas 2/9 patients did not respond to EBP so far (Table 1). Clinical follow-up is available in all patients, whereas 6/9 had good clinical outcome. Two patients responded insufficiently and one patient did not respond at all to EBP. In 3/9 patients, clinical follow-up is limited due to a short follow-up period. Our preliminary data suggest that more than one patch is required for treatment in most cases.

This study raises awareness of the presence of sacral dural leaks in SIH patients and may reduce the number of SLEC-positive patients in whom the site of the leak has not been previously identified. Moreover, SLEC might be overlooked in patients with a subtle sacral leak (as in Fig. 3a), while falsely assuming an underlying CSF-venous fistula.

Our study has the limitation of a retrospective evaluation. Second, the number of cases evaluated in this study is low. Third, inhomogeneous diagnostic work-up was performed (not every patient received further diagnostics such as DSM or CTM). Fourth, T2-MRM has been used to confirm the sacral origin of the leak (in cases where the SLEC was localized exclusively at the sacral level) but has the disadvantage of not being dynamic. On the other hand, it is our experience that epidural fluid is always present at the site of dural tear on spinal MRI and never exclusively distant from the rupture site. Fifth, images were reviewed unblinded and by consensus rather than independently. Sixth, imaging and clinical follow-up after EBP or CT-guided fibrin patch remain incomplete to date.

## Conclusion

Sacral dural leaks were found to occur in 6% of SIH patients in this study and represent another cause of SIH that has been underreported. In contrast to case reports in the literature, we demonstrated that sacral dural tears can occur without meningeal cysts or trauma. Spinal heavily T2-weighted MR images should be implemented in routine SIH imaging covering the entire sacrum to avoid false negative work-up. Epidural lumbar blood patch with the patient in the reverse Trendelenburg position may be a useful therapeutic approach for these patients.

## Abbreviations

CTM: Computed tomography myelography
DSM: Digital subtraction myelography
EBP: Epidural blood patch
MRM: Magnetic resonance myelography
SIH: Spontaneous intracranial hypotension
SLEC: Spinal longitudinal epidural CSF collection
T2-MRM: Heavily T2-weighted MR myelography
CAQ: Certificate of added qualification
